# Enhanced Antitumor Efficacy of an Oncolytic Herpes Simplex Virus Expressing an Endostatin–Angiostatin Fusion Gene in Human Glioblastoma Stem Cell Xenografts

**DOI:** 10.1371/journal.pone.0095872

**Published:** 2014-04-22

**Authors:** Guobin Zhang, Guishan Jin, Xiutao Nie, Ruifang Mi, Guidong Zhu, William Jia, Fusheng Liu

**Affiliations:** 1 Department of Neurosurgery, Beijing Tiantan Hospital affiliated to Capital Medical University, Beijing, P.R. China; 2 Brain Tumor Research Center, Beijing Neurosurgical Institute, Beijing Tiantan Hospital affiliated to Capital Medical University, Beijing, P.R. China; 3 Department of Neurosurgery, Rizhao City People’s Hospital, Shandong, P.R. China; 4 Brain Research Center, Department of Surgery, Faculty of Medicine, University of British Columbia, Vancouver, Canada; Cincinnati Childrens Hospital Medical Center, United States of America

## Abstract

Viruses have demonstrated strong potential for the therapeutic targeting of glioblastoma stem cells (GSCs). In this study, the use of a herpes simplex virus carrying endostatin–angiostatin (VAE) as a novel therapeutic targeting strategy for glioblastoma-derived cancer stem cells was investigated. We isolated six stable GSC-enriched cultures from 36 human glioblastoma specimens and selected one of the stable GSCs lines for establishing GSC-carrying orthotopic nude mouse models. The following results were obtained: (a) VAE rapidly proliferated in GSCs and expressed endo–angio in vitro and in vivo 48 h and 10 d after infection, respectively; (b) compared with the control gliomas treated with rHSV or Endostar, the subcutaneous gliomas derived from the GSCs showed a significant reduction in microvessel density after VAE treatment; (c) compared with the control, a significant improvement was observed in the length of the survival of mice with intracranial and subcutaneous gliomas treated with VAE; (d) MRI analysis showed that the tumor volumes of the intracranial gliomas generated by GSCs remarkably decreased after 10 d of VAE treatment compared with the controls. In conclusion, VAE demonstrated oncolytic therapeutic efficacy in animal models of human GSCs and expressed an endostatin–angiostatin fusion gene, which enhanced antitumor efficacy most likely by restricting tumor microvasculature development.

## Introduction

Glioblastoma is the most common malignant form of primary brain tumor. Glioblastomas rarely spread outside the nervous system, but they infiltrate crucial structures of the brain, which impedes curative surgical resection. Radiotherapy and chemotherapy only offer modest benefits and remain essentially palliative. The overall median length of patient survival is less than 15 months [1]. Thus, most patients with glioblastomas are unlikely to be cured with existing treatments. This condition results in the urgent need to seek new approaches that will improve the curative effect of current therapies.

Recent research suggests that a fraction of cells in glioblastomas have “stem cell-like” characteristics [2], [3], [4], [5]. These tumor-initiating cells have low proliferative rates, high capacity for self-renewal, a propensity to differentiate into actively proliferating tumor cells, and a resistance to chemotherapy or radiotherapy. In addition, these cells are often characterized by a high expression of the stem cell surface marker CD133. Existing research shows that CD133-positive glioblastoma stem cells (GSCs) contribute to tumorigenesis and recurrence [6].

The latest evidence suggests that glioblastoma cells may develop a special relationship with the surrounding vasculature, and the brain tumor microvasculature forms a niche for the maintenance of cancer stem cells (CSCs) [7], [8], [9]. These niches consist of GSCs, ordinary glioma cells, normal host cells, and matrix components [9], [10], [11]. These niches receive nutrition from the microvasculature and transmit intercellular signals [7], [9]. In addition to regulating stem cell proliferation and cell fate decisions, niches shield stem cells from environmental adversity [12], [13]. Thus, vascular niches possibly protect brain CSCs from chemo- and radiotherapies, which enable these cells to reform tumor masses following traditional therapies.

The lack of effective therapies for glioblastomas has prompted the development of novel experimental approaches that utilize oncolytic viruses (OV) combined with gene therapy. OV, either naturally occurring or developed through genetic engineering, are being investigated as oncolytic agents for cancer treatment due to their ability to specifically infect and lyse neoplastic cells while sparing normal cells [14], [15], [16], [17]. The use of transgenic technology facilitates the creation of OV vectors, which deliver therapeutic anti-angiogenic transgenes. This process augments the efficacy of these virus vectors [18], [19], [20]. Genetically engineered OV with anti-angiogenic factors could disrupt the vascular niche, which would expose GSCs and the remaining tumor cells to the oncolytic effects of virotherapy. Furthermore, evidence indicates that a significant portion of the vascular endothelium has a neoplastic origin [21], [22], [23]. Thus, genetically engineered OV could block angiogenesis via the targeted eradication of GSCs, thus improving therapeutic efficacy.

Endostatin and angiostatin, which are potent angiogenesis inhibitors, specifically inhibit the secretion of vascular endothelial cell and fibroblast growth factors. This process inhibits the proliferation of vascular endothelial cells but spares non-endothelial cells. Experiments have shown that endostatin and angiostatin can be used to treat Lewis lung carcinoma, breast cancer, and bladder cancer [24]. Qi Zhan et al. [25] reported that Endostar, a novel recombinant human endostatin, could block VEGF-mediated angiogenesis and has potential effects on human glioma xenografts in nude mice. In addition, these tumors do not develop drug resistance when treated with a potent angiogenesis inhibitor. Based on previous research [26], we used an OV as a vector to target GSC niches and to express a fusion protein consisting of these angiogenesis inhibitors (endo–angio). This virus directly kills cancer cells because of its oncolytic character, and inhibits angiogenesis via viral replication, thus destroying the formation of vascular niches and improving therapeutic efficacy. The use of OV to kill GSCs has been previously reported [17], [27], but the therapeutic efficacy of combining the endostatin–angiostatin fusion gene delivery with oncolytic viral therapy has not been investigated. In the present study, the delivery of the endostatin-angiostatin fusion gene into established GSC-derived tumors has an anti-tumor effect. Overall, our data support the further development of VAE for possible viral gene therapy.

## Materials and Methods

### Ethics Statement

All patients provided written informed consent for the current study, and the study was approved by the Medical Ethics Committee of Beijing Tiantan Hospital, Capital Medical University. The animal research was carried out in strict accordance with the recommendations in the Guide for the Care and Use of Laboratory Animals of the Beijing Tiantan Hospital, Capital Medical University. The protocol was approved by the Animal Experiments Ethics Committee of the Beijing Tiantan Hospital, Capital Medical University. All surgery was performed under 10% chloral hydrate anesthesia, and all efforts were made to minimize suffering.

### Culture and Identification of Primary GSCs

The surgical glioblastoma multiforme (GBM) specimens were obtained from consenting patients at the Beijing Tiantan Hospital, Capital Medical University. The method of primary culture was as described previously [26]. We established six stable GSC lines from 36 surgical specimens that could be passaged for more than 2 months. Then, we selected one of the stable GSCs lines, which could proliferate stably for the subsequent experiments to avoid experimental errors coming from using different GSCs lines. We examined the expression of the CD133 antigen, a putative brain tumor stem-like cell marker, to verify that these cells were GSCs. The cells were differentiated by removing the mitogens and supplementing them with 10% FBS to examine the ability of the cells to create various neural cell lineages. The immunocytochemical analyses of these differentiated cells revealed cells that were positive for the human GFAP, neurofilament protein (NF) and oligodendroglioma myelin-associated glycoprotein (Ompg). These data indicate that the observed cell spheres were human brain GSCs that retained the potential for self-renewal and multi-directional differentiation. CD133, GFAP, NF and Ompg were detected by immunocytochemistry as described previously [26].

### Virus and Infection Studies

The HSV-1 F strain and Vero cells were purchased from ATCC. The oncolytic VAE viruses (γ34.5−, ICP6−, endo–angio+) and r-HSV-1 (γ34.5−, ICP6−; used as a control virus) were constructed by Dr. Willam Jia. The structures of the virus have been previously described [26]. The r-HSV-1 genome consists of long and short unique regions (UL and US), each bounded by terminal (T) and internal (I) repeat regions (RL and RS). The virus was engineered from the wild-type HSV-1 strain F by deleting 1 kb within both copies of the γ34.5 gene. VAE, an oncolytic r-HSV virus, was derived from r-HSV by inserting the endo–angio fusion gene into the ICP6 coding region. This virus was produced in Vero cellsas previously described [28]. The final experimental grade virus preparation was titrated for infectivity, tested for contamination, and stored at −70°C in identical pairs of vials. Each vial contained the virus at the appropriate concentration (10^7^ plaque-forming units, p.f.u.) in a 1 ml total volume of human serum albumin (HSA, 0.5% to 1.0%) in isotonic PBS. The virus was titrated using standard methods in BHK21/C13 cells. Viral titers were expressed as the concentration (p.f.u.) compared with the TCID50 values.

### Viral Cytotoxicity Assay

The GSC spheres were dissociated, and then the cells were resuspended at 2×10^6^ cells/ml and infected with VAE, r-HSV, or PBS at a multiplicity of infection (MOI) of 0.2 for 60 min at 37°C. After centrifugation to remove any un-adsorbed virus, the cells were seeded in 96-well plates at 1×10^4^ cells per well in a serum-free medium. GSC viability was determined every 12 h using an MTS assay as described previously [26]. Similar to our previous study [26], VAE has a similar effect to that of r-HSV-1 in terms of infecting and lysing GSCs.

### In vivo Treatment Studies

Athymic BALB/C-nu mice (male, 20 g to 25 g, 6 weeks to 8 weeks old; Cancer Institute, Chinese Academy of Medical Sciences, Beijing, China) were used for all studies. To facilitate the intracranial tumor studies, the mice were anesthetized via an intraperitoneal injection of 10% chloral hydrate (200 mg/kg). The anesthetized animals were placed in a stereotactic apparatus, and a bur hole was drilled 2 mm lateral to the bregma to a depth of 3 mm. The anesthetized mice (n = 40) were divided evenly into four groups(10/group) and stereotactically inoculated intratumorally with 5 µL of rHSV-1 (5×10^4^ p.f.u.), VAE (5×10^4^ p.f.u.), PBS (1x), or Endostar (5 mg/kg, Simcere, China) 10 days after the tumor cell was implanted with dissociated GBM-SCs (1×10^5^ cells). Coordinates similar to those used for tumor cell implantation were used in the inoculation. The animals were observed daily and were euthanized at the indicated time points or when they showed signs of morbidity (anorexia, movement difficulty, or weight loss of more than 20%).

To facilitate the subcutaneous studies, GBM-SCs (10^6^ cells) were subcutaneously implanted into the rear flank of mice and allowed to grow until they reached approximately 100 mm^3^ in size (measured by vernier caliper). The mice (n = 40) were evenly distributed among four treatment groups (10/group) to enable each group to receive tumor-bearing mice with similar average tumor sizes. Only the control group was injected with PBS (10 µL). One treatment group was injected with Endostar (10 µL). Two treatment groups each were injected with 10 µL of either VAE or rHSV-1 at 1×10^5^ p.f.u. Tumor volumes were measured as indicated, and the mice were euthanized when tumor volumes exceeded 2000 mm^3^ (measured by vernier caliper) or when >20% of their body mass was lost (measured by electronic balance). Tumor growth >20% in mice is deemed progressive. Mice that exhibited 20% reduction in tumor volume after the last treatment were considered responders, whereas mice with tumors that remained within 20% of the original size after the treatment were deemed to have stable diseases.

### MRI Scanning

The MRI scanning of gliomas was conducted using a Siemens 3.0 T MR System 10, 20, 30, and 40 d after inoculation. T1WI and T2WI coronal and horizontal images were acquired from both unenhanced and enhanced scans under the following conditions: slice thickness = 3 mm, interval = 0.3 mm, matrix = 196×160 pixels, NEX = 2, T1WI: SE array, TR 450 ms, TE 8.6 ms and SL = 3 mm, T2WI: TR 5020 ms, TE 100 ms, and SL = 3 mm. The largest areas in the horizontal and coronal planes were analyzed, and the maximum anterior and posterior diameter (L), width (W), and height (H) were used to calculate the tumor volume (V) with the following formula: V = (1/6×π×L×W×H) mm^3^.

### Immunohistochemical Analysis

Immunohistochemical staining was conducted on tumors that had been fixed in 4% paraformaldehyde in 0.1M phosphate buffer (pH = 7.4), dehydrated in 30% sucrose at 4°C, embedded in OCT and frozen at −80°C. Each tumor section was randomly divided into two to four pieces, and 5 µm sections from each piece were stained with goat anti-CD31 (1∶100, Santa Cruz, USA) to visualize the endothelial cells lining the blood vessels (n = 10 mice/group). The three most vascularized areas within the tumor (“hot spots”) were chosen at low magnification, and the vessels were counted from a representative high-magnification (×200) field in each view field. Single immunoreactive endothelial cells or endothelial cell clusters that were separated from other microvessels were counted as individual microvessels. The mean microvessel density (MVD) was calculated as the average count/view field. Vessels at the periphery of the tumor were not included in the MVD counts. The MVDs for each therapy group were then averaged (n = 2 to 4 sections/tumor, and n = 10 tumors/group) to obtain the final count ± SD.

### Reverse Transcription–polymerase Chain Reaction (RT–PCR) Analysis

GSCs infected by VAE, r-HSV-1, or PBS were collected 48 h post-infection. Orthotopically implanted tumors injected with VAE, r-HSV-1, Endostar, or PBS were collected 10 d post-injection. The total RNA from the GSCs and tumors was extracted using Trizol (JCTX, China), and cDNA was obtained using a PrimeScript TM RT–PCR Kit (TaKaRa, Japan). The primers (Invitrogen, USA) had the following sequences: endo–angio: 5′-ACGCATCTTCTCCTTTGACGG-3′ (sense) and 5′-GTCCCTCTGTAGTTCTTTCCATT-3′ (antisense); GAPDH: 5′-GAGTCAACGGATTTGGTCGT-3′ (sense) and 5′-TTGATTTTGGAGGGATCTCG-3′ (antisense). The objective gene fragment and the reference gene were co-amplified. The PCR analysis of endo–angio was performed according to the following conditions: denaturation at 94°C for 2 min, followed by 40 cycles of denaturation for 45 s at 94°C; annealing for 45 s at 58°C; extension for 1 min at 72°C; and extension for 5 min at 72°C. Finally, 5 µl of each PCR product were analyzed via 1.5% agarose gel electrophoresis with ethidium bromide staining and visualization using a UV light transilluminator (Bio Spectrum 500 Imaging System, CA, USA). The lengths of the endo–angio and GAPDH fragments were 330 and 238 bp, respectively.

Image J image processing software (National Institutes of Health, USA) was used for analysis. The gray values of endo–angio and GAPDH were obtained. The relative content of endo–angio mRNA was calculated as follows using GAPDH as the reference: endo–angio mRNA relative content = endo–angio mRNA gray value/GAPDH mRNA gray value.

### Western Blot Analysis

GSCs and medium infected with VAE, r-HSV-1, or PBS were collected 48 h post-infection and orthotopically implanted tumors injected with VAE, r-HSV-1, Endostar, or PBS at 10 d post-injection. The proteins were extracted using a protein extraction kit (Applygen Technologies Inc., China). The protein concentrations were measured using the Bradford method. The proteins were then denatured in SDS–polyacrylamide gel electrophoresis (PAGE) sample loading buffer at 100°C for 10 min and then subsequently separated by SDS–PAGE and transferred onto nitrocellulose filter membranes. The membranes were incubated in TBST containing 5% blocking protein and 0.05% Tween-20 for 60 min. The membranes were immunoblotted overnight at 4°C using the anti-endostatin monoclonal antibody (Millipore, USA) and/or a β-actin antibody (Gene Tex, USA) at approximately 1∶1000 dilution in blocking buffer. The membranes were incubated in goat anti-mouse HRP-conjugated secondary antibody (NeoBioscience, China) at 1∶2000 for 2 h at room temperature after being washed three times in TBST. The protein bands were visualized using enhanced chemiluminescence detection reagents (Applygen Technologies Inc., China).

The Image J image processing software (National Institutes of Health, USA) was also used to analyze the western blot bands. The gray values for endo–angio and β-actin were obtained. The relative content of the endo–angio fusion protein was calculated as follows using β-actin as the reference: endo–angio fusion protein relative content = endo–angio gray value/β-actin gray value.

### Statistical Analysis

All data were expressed as the mean ± SD. All statistical analyses were conducted using SPSS 17.0 statistical software. The Kaplan–Meier curves were compared using the log rank test. Student’s t-tests were used to analyze all other data and a probability value of p<0.05 was considered to be statistically significant.

## Results

### In vitro and in vivo Expression of the Endo–angio Fusion Gene by VAE

**Figure 1:**
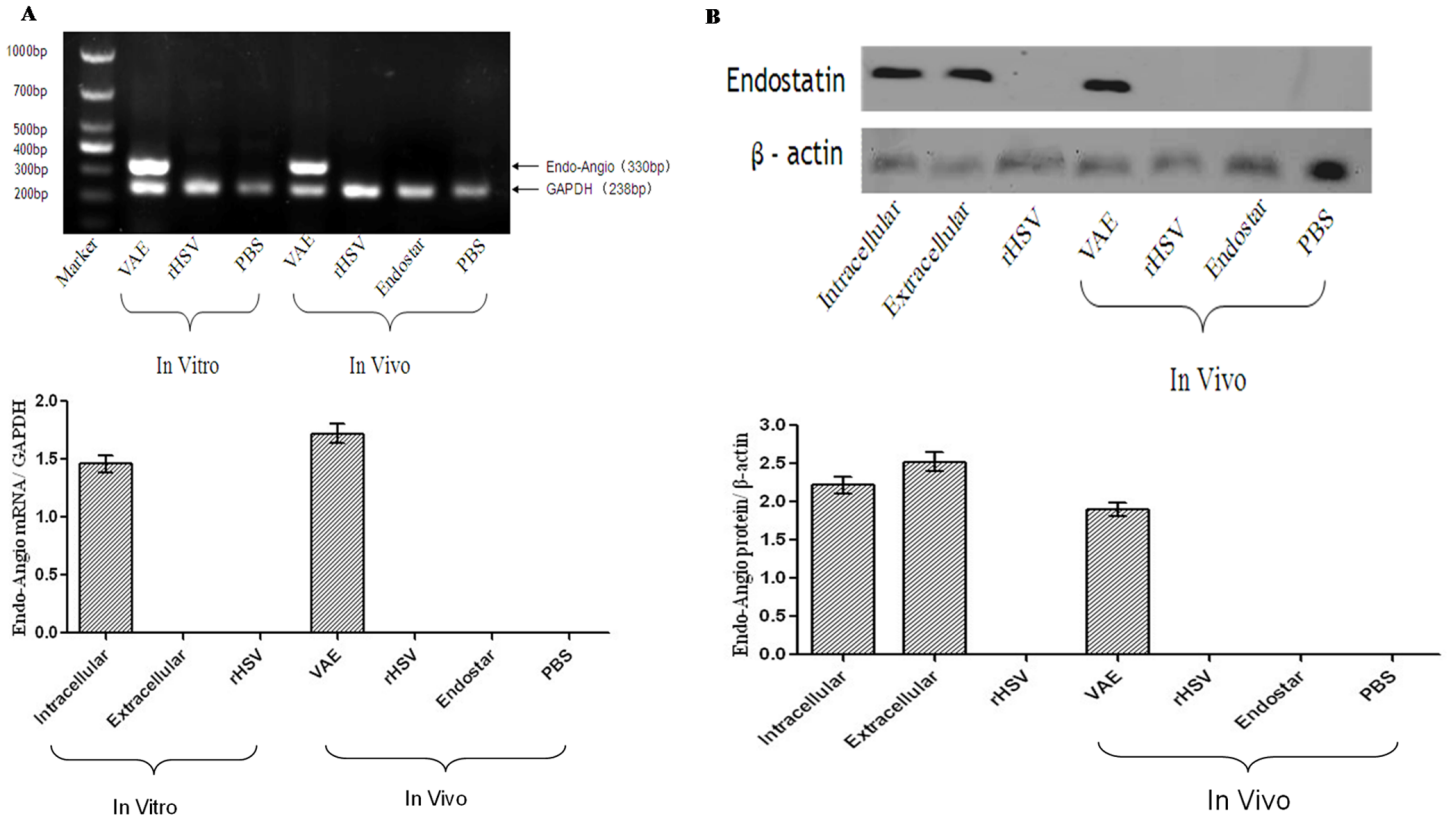
Expression of the exogenous endo–angio fusion gene and the activity of the fusion protein in vitro and in vivo. (A) RT–PCR results indicated that a significant induction of endo–angio mRNA expression existed after VAE infection in vitro and in vivo. By contrast, only the internal standard control, GAPDH, could be detected in the r-HSV-, Endostar-, and PBS-treated groups. (B) Cell lysates and ECM were harvested 48 h after infection, and the orthotopically implanted tumors were harvested 10 d after injection, as described in the Materials and Methods section. The temporal pattern of the expression of endo–angio was investigated via western blot analysis of cell lysates and ECM for the in vitro experiments, and proteins extracted from the tumors for the in vivo experiments. Western blotting results indicated that a 58 kDa fusion protein recognized by the endostatin antibody was present in the VAE group, but not in the r-HSV group or the Endostar group. β-actin was detectable in all samples.

We detected the expression of the endo–angio fusion gene in GSCs infected with VAE in vitro and in vivo using RT–PCR. RT–PCR results revealed significant endo–angio mRNA expression following VAE infection. By contrast, the in vitro r-HSV and PBS groups and the in vivo r-HSV, PBS, and Endostar groups did not express the endo–angio fusion gene. Glyceraldehyde-3-phosphate dehydrogenase (GAPDH) was used for the internal standard (Fig. 1A).

### In vitro and in vivo Production of the Endo–angio Fusion Protein by VAE

Cell lysates and secreted extracellular medium (ECMs) from GSCs infected with r-HSV-1 or VAE were harvested 48 h post-infection to confirm the production of endostatin and angiostatin in vitro. The expression of the endo–angio fusion protein was examined via western blot analysis. The presence of the endo–angio fusion protein was detected in cell lysates and secreted ECMs 48 h after VAE infection of cells in vitro. Mice with established subcutaneous tumors (derived from GSCs) were treated with a single dose of 1×10^5^ p.f.u. VAE via direct intratumoral injection to confirm the production of the endo–angio fusion protein in vivo. The mice were anesthetized with 10% chloral hydrate 10 d after OV therapy, and the harvested tumors were analyzed for endo–angio fusion protein expression by western blot analysis. The molecular weights of endostatin and angiostatin were 20 and 38 kDa, respectively. Thus, the fusion protein was expected to have a molecular weight of 58 kDa. In our experiment, Western blot analysis revealed a band of approximately 58 kDa, indicating that the fusion protein was recognized using this technique. Our western blots indicated that the endo–angio fusion protein was expressed in cells and ECMs infected with VAE and in VAE-treated GSC-derived orthotopic tumors, but not in r-HSV-infected controls. β-actin was used for the internal standard (Fig. 1B).

### VAE Improved Anti-tumor Efficacy against Subcutaneous and Intracranial Gliomas Compared with r-HSV-1

**Figure 2:**
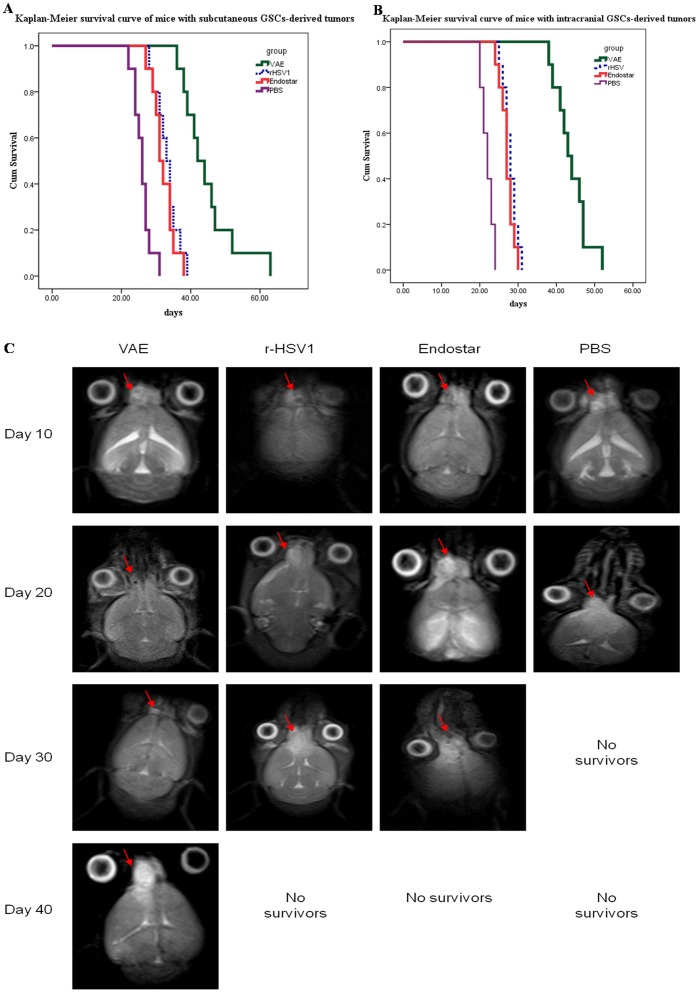
Increased anti-tumor efficacy of VAE compared with r-HSV, Endostar, and PBS. (A) Kaplan–Meier survival curves of mice with subcutaneous GSC-derived tumors treated with PBS, Endostar, r-HSV, or VAE. Mice with subcutaneous GSCs-derived tumors (range = 100 mm^3^ to 200 mm^3^) were treated with PBS, Endostar, or 1×10^7^ plaque-forming units (p.f.u.) of rHSV or VAE on day 10 (n = 10/group) via direct intratumoral injection. The mice were closely monitored for tumor growth and were killed when their tumors reached 2000 mm^3^ or when they lost >20% of their body weight. Mice treated with VAE showed significant improvement in survival compared with mice treated with r-HSV or Endostar (P<0.001). (B) Kaplan–Meier survival curve for mice with intracranial GSC-derived tumors treated with PBS, Endostar, r-HSV, or VAE. Athymic nude mice bearing intracranial GSC-derived tumors were treated with PBS, Endostar, or 2×10^6^ p.f.u. of r-HSV or VAE 10 d after tumor cell implantation. The mice were then closely monitored for survival. Note the statistically significant improvement in median survival of mice treated with VAE compared with r-HSV- and Endostar-treated mice (P<0.001). (C) In a separate intracranial glioma experiment, mice were treated with VAE, r-HSV, Endostar, or PBS on day 10 after tumor cell implantation and examined on days 10, 20, 30, and 40 via MRI. Representative images of MRI T2-axial sections indicating the changes in tumor size (red arrows) after treatment with VAE, rHSV, Endostar, or PBS are shown. Note that all animals in the PBS-treated group developed large tumors during the treatment period. VAE-treated mice underwent complete remission, and the r-HSV- and Endostar-treated groups exhibited stable disease on day 20. After day 20, residual tumors in the VAE-treated mice regrew, and tumors in r-HSV- and Endostar-treated mice rapidly worsened (see results on day 30).

We first tested the anti-tumor efficacy of VAE in subcutaneous gliomas in athymic nude mice. Mice with subcutaneous tumors (approximately 100 mm^3^) were treated with VAE, r-HSV-1, Endostar, or PBS, and then closely monitored for tumor growth. All mice treated with PBS rapidly progressed after the treatment. A trend toward enhanced survival in mice treated with VAE was noted compared with rHSV, Endostar, or PBS. The r-HSV-, VAE-, and Endostar-treated mice appeared to have tumor necrosis and stable disease. Figure 2A shows the Kaplan–Meier survival curve analysis of mice treated with VAE, r-HSV-1, Endostar, or PBS. A statistically significant increase in the survival of mice (n = 10/group) treated with VAE (median survival = 42 d) was noted compared with those treated with rHSV (median survival = 33 d) (P<0.001), Endostar (median survival = 31 d) (P<0.001), and PBS (median survival = 26 d) (P<0.001).

We compared the anti-tumor efficacy of VAE with that of rHSV, Endostar, or PBS in mice bearing intracranial gliomas. Ten days after the tumor implantation, the mice were treated with a single dose of VAE, r-HSV-1, Endostar, or PBS via direct intratumoral injection. The survival of the mice in each group (n = 10/group) was analyzed using Kaplan–Meier curves (Fig. 2B). The control mice treated with PBS died of tumor burden, with a median survival of 22 d. The mice treated with VAE exhibited a significant increase in median survival compared with rHSV-treated mice (median survival = 43 and 28 d, respectively; P<0.001) and Endostar (median survival = 43 and 27 d, respectively; P<0.001).

In a separate experiment, mice with intracranial gliomas treated with VAE, rHSV, Endostar, or PBS were examined by MRI on days 10, 20, 30, and 40 following GSC implantation. The mice received interventional measures on day 10 after GSC implantation. On day 20, the VAE-treated group had undergone complete remission based on the MRI (Fig. 2C), whereas the rHSV- and Endostar-treated groups exhibited stable diseases. All animals in the PBS-treated group developed large tumors during the treatment period (Fig. 2C). The mice in the rHSV- and Endostar-treated groups showed significant tumor progression and exhibited symptoms of tumor burden, whereas the VAE-treated mice showed a regrowth of the residual disease on day 30. This finding suggested that VAE treatment of intracranial tumors suppressed the regrowth of residual tumors after oncolysis compared with rHSV and Endostar treatment.

### Changes in MVD in the Established GSC-derived Tumors Treated with VAE

**Figure 3:**
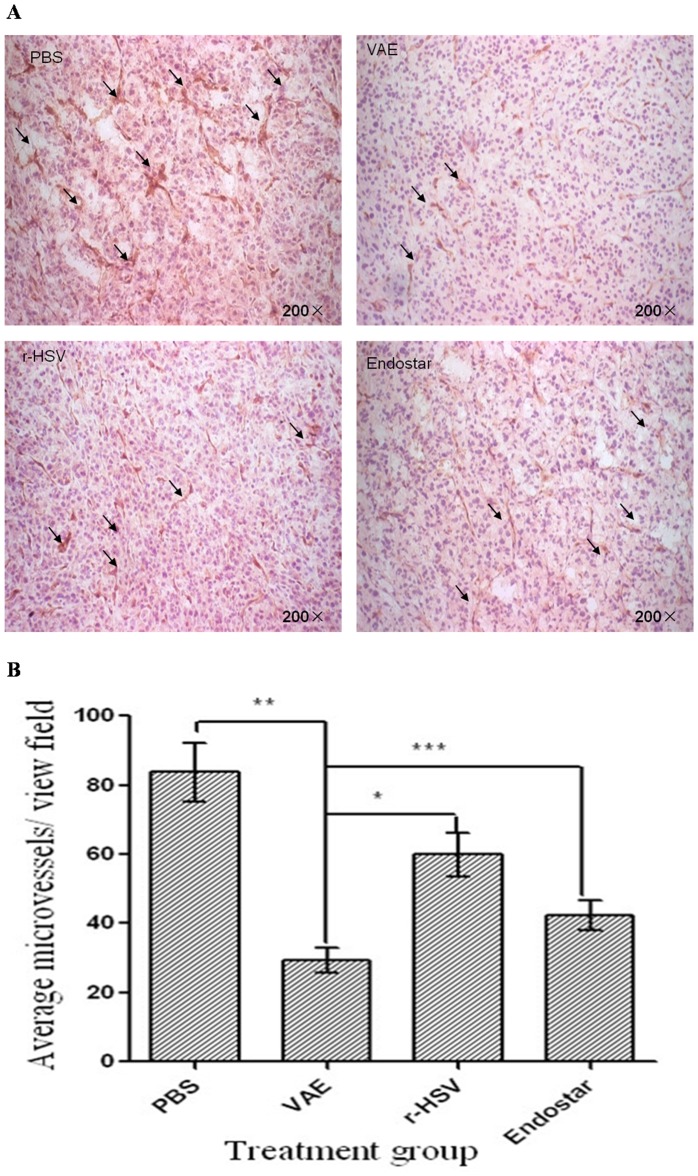
Reduced MVD and perfusion in animals treated with VAE compared with rHSV, Endostar, and PBS. Mice with subcutaneous GSC-derived tumors were treated with a single dose (1×10^7^ plaque-forming units) of rHSV, VAE, PBS, or Endostar 10 d after tumor cell implantation. (A) Representative pictures of immunohistochemical staining for CD31 in tumors isolated from mice 10 d after therapy with PBS, VAE, r-HSV, or Endostar. Scale bar: 10 µm. (B) The quantification of MVD in tumors treated with PBS, VAE, r-HSV, or Endostar. Data are shown as the mean MVD ± SEM for each group (n = 2 to 4 sections/tumor and n = 10 tumors/group). Note the significant difference in MVD for VAE versus r-HSV (*P<0.001), PBS versus VAE (**P<0.001), and Endostar versus VAE (***P<0.001).

We compared the MVD of tumors treated with VAE, r-HSV-1, Endostar, and PBS to determine whether the VAE treatment of tumors resulted in anti-angiogenesis and decreased tumor regrowth. Ten days after subcutaneous GSC implantation, the mice were treated with a single intratumoral injection of VAE, r-HSV-1, Endostar, or PBS. Ten days after the therapy, the tumors were harvested and sections were stained for CD31, a vascular marker, to visualize the vessels (Fig. 3A). Microvessel count revealed a statistically significant reduction in MVD for tumors treated with VAE compared with r-HSV-1, Endostar, and PBS (P<0.0001, P<0.0001, and P<0.0001, respectively) (Fig. 3B).

## Discussion

Recent studies indicated that GSCs and their vascular niches are the roots of proliferation and recurrence in gliomas [8], [9], [13], [29]. Thus, therapies designed to attack GSCs and vascular niches have become hot topics. Recombinant oncolytic viral therapy is a novel approach for glioma treatment that has produced promising results in two fields of emerging anti-tumor therapy: gene therapy and virotherapy [30]. Tumor cells are directly targeted for destruction by the oncolytic activity of the recombinant oncolytic viruses; thus, normal neural tissues are not harmed [15], [26]. Although the effect of using oncolytic viruses carrying exogenous genes in cancer therapy is controversial, our research demonstrates the efficacy of VAE (*γ34.5^–^*, *ICP6^–^*, *endo–angio* ^+^) in GSCs. This oncolytic virus lacks both copies of the *γ34.5* gene and has a gene-disrupting insertion of the endo–angio fusion gene within the viral *U_L_39* locus that encodes the ICP6 gene. The strategy for the creation of VAE has been reported in our previous study [26] which was the first to investigate the potential of endo–angio fusion gene delivery as a therapeutic modality in GSC-derived tumors in vivo. Our results indicate the following: (a) VAE rapidly proliferated in GSCs and expressed endo–angio 48 h and 10 d after infection (in vitro and in vivo, respectively); (b) after treatment with VAE, subcutaneous gliomas derived from GSCs indicated significant reductions in MVD compared with controls treated with rHSV or Endostar; (c) a significant improvement in the survival time of mice with intracranial and subcutaneous gliomas treated with VAE was noted compared with rHSV and Endostar; and (d) MRI indicated that the tumor volumes of intracranial gliomas derived from GSCs remarkably decreased 10 d after treatment with VAE compared with rHSV and Endostar. The conclusions drawn from the results are restricted to the particular single GSC examined. However, the significance obviously is there for antitumor efficacy of VAE. This method makes full use of the advantages of the oncolytic properties of VAE and its ability to express exogenous genes. Moreover, its usefulness as a therapy confirms the importance of targeting GSCs and their vascular niche.

We have isolated stem cell-like cells from human GBM and have shown their capacity for self-renewal, their ability to differentiate into multiple cell types, and their efficient tumorigenicity in an orthotopic nude mouse model. GSCs are the foundations of our experiment that allow us to perform in vitro investigations to test their susceptibility to VAE, as well as in vivo therapeutic studies. Thus, in this study, we used a defined culture medium to select and expand the cells as stable cultures instead of selecting cells using a specific marker, such as CD133. Early studies suggested that only CD133+ cells could form tumors in the brains of immunocompromised mice [2]. However, recent studies demonstrated the relative lack of specificity of this marker and indicated that CD133-populations can also form tumors in vivo [31]. Therefore, we used cultures based on neurospheres that contained both CD133+ and CD133- cells, thus avoiding the possibility of omitting potential tumor-initiating cells.

Hiroaki et al. reported that primary GSC culture-derived tumors are highly invasive and progress to well-delineated tumor masses with increased vascularity [17]. This finding is of great value for glioma research because the readily available mouse models of human glioma, such as U87 xenografts, do not display invasiveness, an important trait of human GBM [32]. Thus, our orthotopic model using GSCs could better represent human disease.

Using this orthotopic model based on GSCs, we have shown the therapeutic efficacy of a single intratumoral inoculation of VAE. Intratumoral treatment with VAE resulted in an extensive infection of the migrating tumor cells and a subsequent increase in survival. The experimental treatment of U87-derived tumors with RAMBO was recently reported. In this study, a significant spread of the vector was observed, along with a significant survival benefit and a decrease in the role of angiogenesis [19]. Although the model used in this study is different from ours, our findings revealed similar results.

Phase I/II clinical testing of OV therapy has demonstrated the relative safety of this approach in human patients, and future large randomized phase III clinical trials that will evaluate the therapeutic efficacy of this approach are being planned [33], [34], [35]. One of the major limitations of OV therapy is the generation of rapid, innate immune responses initiated in tumors upon infection, which are accompanied by the secretion of several pro-angiogenic factors that can induce angiogenesis and tumor recurrence [36], [37]. Kurozumi et al. [38] reported that the reduction of tumoral blood vessel density by a single dose of an angiostatic cRGD peptide prior to oncolytic HSV-1 treatment reduced OV-induced hyperpermeability and tumor inflammation and prolonged tumoral viral propagation, thus enhancing the anti-tumor efficacy of OV. However, some OVs display an innate ability to infect ECs in tumors, while sparing those in normal vessels [39]. This induced neutrophil infiltration, leading to microclot formation within the tumor-associated vasculature, resulting in a large bystander effect of cell death within the tumor [40]. This provides a useful platform for the further development of OVs armed with therapeutic transgenes in the future. In the present study, we showed that treatment with VAE, an engineered oncolytic HSV-1 expressing an endo–angio fusion gene, exerted significantly greater anti-tumor effects and a reduction in tumor MVD compared with r-HSV-1 or Endostar treatment alone. The increase in the anti-tumor efficacy of VAE may be due to its ability to kill tumor cells and decrease angiogenesis. In future studies, we will elucidate whether the expression of the endo–angio fusion gene also affects intratumoral VAE propagation and antiviral immune responses in the tumor. In addition, our analysis of MVD reduction was restricted to subcutaneous gliomas. Future studies are required to determine whether microvasculature is also reduced in the intracranial gliomas treated with VAE.

The brain tumor microvasculature includes specific niche microenvironments that are critical for the maintenance of GSC self-renewal and multipotency [9]. Although multiple studies have begun to delineate the relationship between GSCs and vascular cells, the precise nature of the niche has yet to be fully characterized [9], [41], [42]. The GSCs and the microvascular niche have a symbiotic relationship. GSCs secrete angiogenic factors that promote the recruitment and formation of tumor blood vessels [6]. Soluble factors released from endothelial cells contribute to GSC maintenance and proliferation [41]. The importance of this vascular niche has been highlighted in recent studies reporting a decrease in the number of GSCs following treatment with angiogenesis inhibitors [7]. Recent studies have described a new mechanism for tumor vasculogenesis, that is, GSCs are able to differentiate into functional endothelial cells [21], [22], [23]. In our study, we examined the effect of VAE on GSC-derived glioma xenografts. We showed that VAE had significantly greater antitumor effects than previously employed therapies in both subcutaneous and intracranial animal models. This result could be ascribed to VAE, which expresses an antiangiogenic gene (endo–angio) that disrupts the vascular niche, exposing both GSCs and the remaining tumor cells to oncolytic virus therapy. Meanwhile, functional endothelial cells derived from GSCs could also be destroyed by VAE, which also inhibits glioma angiogenesis, thus generating a vicious cycle between GSC propagation and angiogenesis.

Although a single inoculation of VAE significantly increased the survival of tumor-bearing mice, we did not achieve a full cure, possibly due to the following events: (a) the tumor cells possibly infiltrated the surrounding normal brain, and thus, escaped the effects of VAE; (b) the GSCs possibly secreted several as yet unknown factors that promoted angiogenesis; and (c) the transfection efficiency of VAE was not high enough to destroy all of the target cells. Thus, further improvement of this treatment strategy is needed.

## Conclusion

We have established an orthotopic GSC-derived tumor model that is useful for research into glioma therapies. This study underscores the significance of combining angiostatic strategies with oncolytic viral therapy, and future work with this virus may lead to a new modality for the treatment of cancer.
